# Increases in [IP_3_]_i_ aggravates diastolic [Ca^2+^] and contractile dysfunction in Chagas’ human cardiomyocytes

**DOI:** 10.1371/journal.pntd.0008162

**Published:** 2020-04-10

**Authors:** Alfredo Mijares, Raúl Espinosa, José Adams, José R. Lopez

**Affiliations:** 1 Centro de Biofísica y Bioquímica, Instituto Venezolano de Investigaciones Científicas, Caracas, Venezuela; 2 Departamento de Cardiología, Hospital Miguel Pérez Carreño, Instituto venezolano de los Seguros Sociales, Caracas, Venezuela; 3 Division of Neonatology, Mount Sinai, Medical Center, Miami, FL, United States of America; 4 Department of Research, Mount Sinai, Medical Center, Miami, FL, United States of America; Universidade Federal de Minas Gerais, BRAZIL

## Abstract

Chagas cardiomyopathy is the most severe manifestation of human Chagas disease and represents the major cause of morbidity and mortality in Latin America. We previously demonstrated diastolic Ca^2+^ alterations in cardiomyocytes isolated from Chagas’ patients to different degrees of cardiac dysfunction. In addition, we have found a significant elevation of diastolic [Na^+^]_d_ in Chagas’ cardiomyocytes (FCII>FCI) that was greater than control. Exposure of cardiomyocytes to agents that enhance inositol 1,4,5 trisphosphate (IP_3_) generation or concentration like endothelin (ET-1) or bradykinin (BK), or membrane-permeant myoinositol 1,4,5-trisphosphate hexakis(butyryloxy-methyl) esters (IP_3_BM) caused an elevation in diastolic [Ca^2+^] ([Ca^2+^]_d_) that was always greater in cardiomyocytes from Chagas’ than non- Chagas’ subjects, and the magnitude of the [Ca^2+^]_d_ elevation in Chagas’ cardiomyocytes was related to the degree of cardiac dysfunction. Incubation with xestospongin-C (Xest-C), a membrane-permeable selective blocker of the IP_3_ receptors (IP_3_Rs), significantly reduced [Ca^2+^]_d_ in Chagas’ cardiomyocytes but did not have a significant effect on non-Chagas’ cells. The effects of ET-1, BK, and IP_3_BM on [Ca^2+^]_d_ were not modified by the removal of extracellular [Ca^2+^]_e_. Furthermore, cardiomyocytes from Chagas’ patients had a significant decrease in the sarcoplasmic reticulum (SR) Ca^2+^content compared to control (Control>FCI>FCII), a higher intracellular IP_3_ concentration ([IP_3_]_i_) and markedly depressed contractile properties compared to control cardiomyocytes. These results provide additional and convincing support about the implications of IP_3_ in the pathogenesis of Chagas cardiomyopathy in patients at different stages of chronic infection. Additionally, these findings open the door for novel therapeutic strategies oriented to improve cardiac function and quality of life of individuals suffering from chronic Chagas cardiomyopathy (CC).

## Author summary

Chagas disease, caused by the parasite *Trypanosoma cruzi*, is an endemic disease of Latin-American countries, affecting 10 million people are estimated to be infected with *T*. *cruzi*, and more than 120 million inhabitants are at risk of infection. The parasite is transmitted to humans in a vectorial way by infected triatomines and through other non-vector mechanisms such as the oral route, congenital transmission, organ transplants or blood transfusions. Due to immigration towards non-endemic regions, the disease can spread and affect people around the world via blood transfusions. The pathogenesis of this disease is still unwell understood; we previously demonstrated that cardiomyocytes isolated from Chagas patients have an intracellular Ca^2+^ overload, which appears to be associated with changes in the inositol 1,4,5 trisphosphate (IP_3_) signaling pathway. This study corroborates that human cardiomyocytes isolated from Chagas’ patients have an increase in [Ca^2+^]_d_ and a partial membrane potential depolarization, which corresponds with the degree of cardiac dysfunction determined by the NYHA classification (23). In this report, we showed, for the first time, that IP_3_R activators, e.g., IP_3_BM, ET-1, and BK-induced a more significant elevation of [Ca^2+^]_d_ in Chagas’ compared to non-Chagas’ human cardiomyocytes, which was not modified by the removal of [Ca^2+^]_e_. Additionally, these findings open the door for novel therapeutic strategies oriented to improve cardiac function and quality of life of individuals suffering from chronic Chagas cardiomyopathy (CC).

## Introduction

Chagas disease (American trypanosomiasis) is caused by the protozoa parasite *Trypanosoma cruzi* (*T*. *cruzi*), which is transmitted to humans by blood-sucking triatomine bugs and by non-vectorial mechanisms, such as contaminated blood transfusion, organ transplantation, and congenital infection [1, 2]. Chagas disease is a significant public health burden and the leading cause of death and morbidity in Latin American and Caribbean regions [3]. Worldwide, 10 million people are estimated to be infected with *T*. *cruzi*, and more than 120 million inhabitants are at risk of infection [4]. As a neglected disease, Chagas’ disease is associated with malnutrition, poverty, and inadequate sanitation [5], and it is part of a self-propagating cycle of poverty in many endemic regions. Human migrations due to economic hardship, political problems, or both, have spurred an exodus from Chagas-endemic countries to geographical areas where the disease was not endemic [6–9]. Individuals with Chagas disease have been identified in non-endemic countries in Europe, Canada, and the USA [7, 10], and an estimated 300,000 persons are suffering from this disease who live in the US, especially in Texas and along the Gulf coast [11, 12]. Chagas’ disease has become a potentially severe emerging threat to several countries throughout the world.

Chagas' disease is a multifactorial illness that consists of two sequential phases, an initial acute phase, followed by a chronic phase that can be categorized into a cardiac or digestive form [13]. The initial acute phase lasts for about 2 months after infection, and it is limited to a febrile episode, headache, enlarged lymph glands, muscle pain, and abdominal or chest pain [14]. In the chronic phase, 20–40% of the infected patients go on to develop cardiomyopathy or digestive damage (typical enlargement of the esophagus or colon) [15–17]. Chagas cardiomyopathy (CC) is an important form of chronic Chagas' disease which has a high morbidity and mortality and a significant medical and social impact. CC is associated with myocarditis, rhythm disturbances, depressed heart function, congestive failure, thromboembolism, and sudden death [14, 18]. The most important prognostic marker in CC is the severity of myocardial contractile dysfunction [19].

Despite the extensive characterization of the clinical manifestations of CC, the mechanisms underlying the pathogenesis of this disease are still poorly understood. Earlier studies with non-human models [20–22] have shown there is a possible link between Chagas’ infection and alteration in phospholipase-C/phosphoinositide signaling pathway. We recently demonstrated that cardiomyocytes isolated from Chagas patients have an intracellular Ca^2+^ overload, which appears to be associated with changes in the inositol 1,4,5 trisphosphate (IP_3_) signaling pathway [23]. IP_3_ is a second messenger generated by hydrolysis of membrane lipid phosphatidylinositol 4,5-bisphosphate by phospholipase C in response to G protein-coupled receptor activation [24]. Once generated, IP_3_ causes Ca^2+^ release from the sarcoplasmic reticulum (SR) and the nuclear envelope via the IP_3_ receptors (IP_3_Rs) [24]. In the heart, IP_3_Rs are thought to play an important role by modulating Ca^2+^ signals during excitation-contraction coupling (ECC) and cardiac gene expression. IP_3_Rs activation is characterized by increasing action potential amplitude, and spontaneous Ca^2+^ transient frequency, and decreasing resting membrane potential [25–27]. However, the role of IP_3_Rs in cardiac ECC is controversial due to lower expression levels in ventricular cardiomyocytes compared to other cell types [28, 29].

The present study was undertaken to further investigate the involvement of IP_3_ in the diastolic Ca^2+^ and contractile dysfunctions observed in cardiomyocytes isolated from Chagas’ patients.

## Methods

### Ethics statement

Written consent from all patients involved in this study was obtained prior to processing the samples. Invasive cardiac studies were performed after the patient provided written informed consent, and approval was granted by the Bioethics Committee of Hospital Pérez Carreño (No. 073/17), Caracas, Venezuela. Data on human subjects were analyzed anonymously, and clinical investigations have been conducted according to the Declaration of Helsinki.

### Patient’s study population

This study was conducted in 33 Chagas’ patients with CC (see Table 1). Chagas patients had an abnormal electrocardiogram at rest (rhythm disturbance and conduction defects), positive blood culture and enzyme-linked immunosorbent assay (ELISA) for the Chagas disease. None of them had congestive heart failure or ischemic heart disease. Patients were grouped based on the New York Heart Association (NYHA) classification system, which considers the patient's clinical manifestations and risk factors that affect mortality: early (functional class I (FCI), intermediate (functional class II (FCII), and late (functional class III (FCIII). According to the NYHA 18 patients of the Chagas’ patients fell within functional class I (FCI), and 15 patients in FCII, according to the NYHA. Besides, 17 non- Chagas’ subjects (considered as control) with mild mitral stenosis and negative blood culture, and ELISA for Chagas disease served as control (see Table 1). Potential subjects (control or Chagas’ patients) were excluded from the study if they had a history of alcoholism.

**Table 1:** 

	Age (years)	Sex F M		FCI Pts *(n)*	FCII Pts *(n)*	ECG disturbances	Medications	*n*
Chagas’	45±6 Range:36–55	10	23	F:6—M:12	F:4—M:11	Rhythm disturbance and conduction defects (80%)	ACE; BB; DIT; D; ATR	33
Control	36±6 Range: 27–45	7	10	-	-	Atrial fibrillation (20%)	BB; DIT; CCB; AC; ATB; ATR	17

**Abbreviations**: F = Female; M = Male; Pts = patient; FCI = Functional class I; FCII = Functional class II; ECG = electrocardiogram; ACE = Angiotensin converting enzyme inhibitors inhibitor; BB = Beta blockers; DIT = diuretics; D = Digitalis; ATR = Antiarrhythmics; CCB = L-type Ca^2+^ channel blockers; AC = anticoagulant; ATB = Antibiotics. Values are expressed as mean ± SD.

### Endomyocardial biopsy

Left ventricular endomyocardial biopsies were obtained from the Chagas’ patients using fluoroscopic as part of routine evaluation for Chagas patients at the Cardiology Department at Hospital Miguel Perez Carreño (Caracas, Venezuela). The Chagas’ patients were pretreated with aspirin 800 mg twice daily on the day preceding the examination and 800 mg before the procedure to reduce the thromboembolic risk. Biopsies from control subjects were obtained during mitral valve replacement surgery. Although not all patients included in this study were taking medications at that time, those who were stopped their medications 48 h before the endocardial biopsies. Upon removal, the endomyocardial biopsies were immediately immersed in ice-cold, oxygenated, low Ca^2+^- solution supplemented with 2,3-butanedione monoxime (BDM) to prevent Ca^2+^-induced hypercontraction (see solutions). BDM *reduce*s the activity of the myosin ATPase, inhibits Ca^2+^-induced force development [30], and decreases reoxygenation injury [31]. The connective tissue was removed from the biopsy specimens with the aid of a dissecting microscope, and the tissue was cut into small pieces. Calcium tolerant cardiomyocytes were isolated enzymatically following the technique described by Peeters et al. 1995 [32]. The isolated cardiomyocytes were settled for 10 min sequentially in a buffer solution containing 50 μM, 100 μM, 500 μM and 1.8 mM Ca^2+^, and at each step the injured cells (spontaneous contractile activity was discarded). The yield of Ca^2+^-tolerant ventricular cardiomyocytes (rod-shaped) was significantly higher in control samples (75%) than in cardiomyocytes from Chagas’ patients (64% from FCI and 55% from FCII). This difference may due to the increased fibrosis and plasma membrane damage observed in cardiomyocytes from Chagas’ patients [15].

### Criteria for selecting cardiomyocytes

Cardiomyocytes were studied if they had sharp outlines and rod-shaped, clearly visible striations, without developing subsarcolemmal blebs, and showing spontaneous contractile activity in the presence of 1.8 mM extracellular [Ca^2+^]. In some experiments, cell integrity was further determined by the ability of the cardiomyocyte to exclude the dye trypan blue.

### Ca^2+^ and Na^+^-selective microelectrodes

Double-barreled Ca^2+^ and Na^+^ selective microelectrodes were prepared as described previously [33]. Each ion-selective microelectrode was individually calibrated before and after the determination of diastolic Ca^2+^ concentration ([Ca^2+^]_d_) and diastolic Na^+^ concentration ([Na^+^]_d_) as described before [33]. Only those Ca^2+^ selective microelectrodes with a linear relationship between pCa 3 and 7 (Nernstian response 30.5 mV/pCa unit at 37°C, respectively) were used experimentally. The Na^+^ selective microelectrodes gave virtually Nernstian responses at free [Na^+^]_e_ between 100 and 10 mM. However, although at concentrations between 10 and 1 mM [Na^+^]_e_, the microelectrodes had a sub-Nernstian response (40–45 mV), their response was of sufficient amplitude to be able to measure [Na^+^]_d_. The response of the Ca^2+^ and Na^+^-selective microelectrodes were not directly affected by any of the drugs used in the present study.

### Measurements of [Ca^2+^]_d_ in human cardiomyocytes

Within 1–2 h after isolation, human Ca^2+^ tolerant cardiomyocytes were transferred to poly-L-lysine-coated coverslips for 45 minutes in a small Plexiglas chamber filled with normal Tyrode solution containing 20 mM BDM at 37°C. Only rod-shaped cardiomyocytes without any signs of deterioration and spontaneous activity at rest were used for experiments [23, 34]. Cardiomyocytes from control and Chagas’ patients were impalements with the doubled-barreled Ca^2+^ selective microelectrodes with the aid of an inverted microscope fitted with an x10 eyepiece and an x40 oil objective. The potentials from the 3 M KCl barrel -resting membrane potential (Vm)- and the Ca^2+^ barrel (V_CaE_) were recorded via a high-impedance amplifier (model FD-223; WPI, Sarasota, FL). The potential of the voltage microelectrode (Vm) was subtracted electronically from the potential of the Ca^2+^ electrode (V_CaE_) to obtain the differential signal (V_Ca_) representing the resting [Ca^2+^]_d_. Vm and VCa potentials were acquired at a frequency of 1,000 Hz with AxoGraph software (version 4.6; Axon Instruments, Foster City, CA), and stored in a computer for further analysis. Two criteria were used as key elements to accept or to reject individual [Ca^2+^]_d_ measurements performed in cardiomyocytes from control and Chagas’ patients: i) polarize resting membrane potential -more negative than -80 mV in control and more than -75 in Chagas cardiomyocytes- and ii) stable recording potentials for no less than 40 seconds (Vm, V_Ca_).

### Sarcoplasmic reticulum Ca^2+^ content

To estimate the total amount of Ca^2+^ stored in the sarcoplasmic reticulum (SR), control and Chagas’ cardiomyocytes were loaded with 5 μm Fluo-4-AM for 30 min at 37°C. Fluo-4 loaded cardiomyocytes were transferred to a small Plexiglas chamber filled normal Tyrode solution containing 20 mM BDM and placed on Plexiglass chamber on the stage of an inverted microscope equipped with epifluorescence illumination (XCite® Series 120 or Lambda DG4) equipped with a CCD cooled camera (Retiga 2000R or Stanford Photonics 12 bit digital). The excitation wavelength of the argon-ion laser was set to 488 nm, and fluorescence emission was measured at wavelengths >515 nm. The experiments were conducted in a Ca^2+^-free solution to prevent the Ca^2+^ uptake by the SR. The Ca^2+^ transient elicited by 10 mM caffeine (2 min stimulus) was used as an index of the Ca^2+^ content of the SR, which was estimated by taking the area under the curve of the signal induced by caffeine [35]. The experiments were carried out in a blinded fashion to validate our results.

### Determination of cytosolic [IP_3_]

Intracellular [IP_3_] was determined in cardiomyocytes biopsies from control subjects and Chagas’ patients using a competitive radioligand binding assay, as previously described [36]. In brief, ventricular myocytes from control or Chagas’ patients were suspended in normal Tyrode solution maintained at 38°C. Each sample was pre-incubated for 10 min, with 10 mM LiCl to inhibit inositol phosphate metabolism [37]. The tubes were maintained in ice for 20 min, then centrifuged, and the pellet was kept for protein determination by the Lowry method [38]. The supernatant was neutralized to pH 7.0 with 1.5 M KOH containing 60 mM HEPES. The intracellular IP_3_ concentration was determined using the IP_3_ assay kit (Amersham, Arlington Heights, IL) according to the manufacturer’s instructions.

### Cardiomyocyte contractility studies

Contractile properties of Chagas’ and control cardiomyocytes were studied in a custom-designed Perspex chamber with a glass-bottom filled with normal Tyrode solution, using a video-based edge-detection system (IonOptix, Milton, MA). The cardiomyocytes were field stimulated through a pair of platinum electrodes at a frequency of 1 Hz (2 ms pulse duration ~1.5x threshold voltage). Myocyte edges were continuously tracked during contraction and relaxation, displayed as a voltage signal proportional to the changes in myocyte length, and sent to a PC for future analyses of different contraction and relaxation parameters (IonOptix, Milton, Massachusetts). The following parameters were measured: i) diastolic sarcomere length which was determined after a 30-s stimulation (2 ms pulse duration ~1.5x threshold voltage) in quiescent cardiomyocytes; ii) peak shortening (PS), indicative of peak ventricular contractility; iii) maximal velocity of shortening (+dL/dt), indicative of ventricular pressure rise; iv) maximal velocity of relengthening (−dL/dt), indicative of ventricular pressure fall. Only rod-shaped cardiomyocytes with good striation and edges were used. Experiments were conducted at 37°C.

### Solutions

All solutions were made using ultrapure water supplied by a Milli-Q system (Millipore, Bedford, MA). Tyrode solution had the following composition (in mM): NaCl 130, KCl 2.68, CaCl_2_ 1.8, MgCl_2_ 1, NaHCO_3_ 12, NaH_2_PO_4_ 0.4, glucose 5, and pH 7.4. For the conditions where a Ca^2+^-free solution was required, the 2 mM CaCl_2_ was replaced with 2 mM MgCl_2_, and 1 mM EGTA was added. 2,3-butanedione monoxime, endothelin, bradykinin, IP_3_BM, and L-IP_3_PM membrane-permeant esters of IP_3_, xestospongin-C, or caffeine were added to the desired concentration to Tyrode’ solution immediately before use. Cardiomyocytes were perfused with Tyrode’ solution aerated with 95% O_2_ and 5% CO_2_. All experiments were performed at 37 ^o^C.

### Statistical analysis

All values are expressed as mean±SD; ***n*** represents the number of cardiomyocytes (control or Chagas) in which a successful measurement of [Ca^2+^]_d_ was carried out. The area-under-the-curve for the caffeine-induced release of Ca^2+^ from the SR was calculated by the trapezoid rule (GraphPad Prism software 7.0). Statistical analysis was performed using a two-tailed paired and unpaired *t-*test or one-way analysis of variance coupled with either Tukey’s or Dunnett’s *t*-test for multiple measurements to determine significance. Significance was accepted at *p*<0.05 level. Statistical analysis was done using GraphPad Prism 7.03 (GraphPad Software, Inc.).

## Results

### [Ca^2+^]_d_ and [Na^+^]_d_ in cardiomyocytes from Chagas’ patients

**Fig 1:**
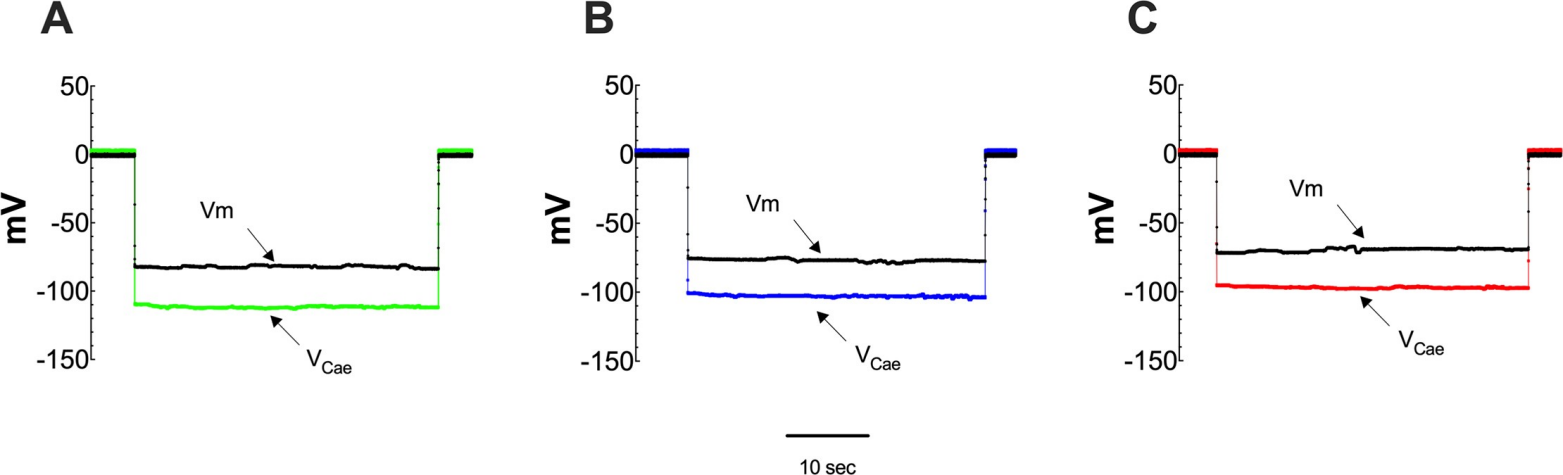
Diastolic [Ca^2+^] is greater in cardiomyocytes from patients suffering from Chagas cardiomyopathy than control. Representative simultaneous measurements of the resting membrane potential (Vm) and diastolic Ca^2+^ concentration ([Ca^2+^]_d_) in cardiomyocytes isolated from control (CTR) and Chagas’ patients (FCI and FCII). **(A)** Recording of Vm = -83 mV and [Ca^2+^]_d_ = 122 nM measured in a control cardiomyocyte; **(B)** Recordings of Vm and [Ca^2+^]_d_ from a cardiomyocyte isolated from Chagas’ patient FCI (Vm = -75 mV and [Ca^2+^]_d_ = 263 nM); **(C)** Recordings of Vm and [Ca^2+^]_d_ from a cardiomyocyte isolated from Chagas’ patient FCII (Vm = -72 mV and [Ca^2+^]_d_ = 364 nM).

**Fig 2:**
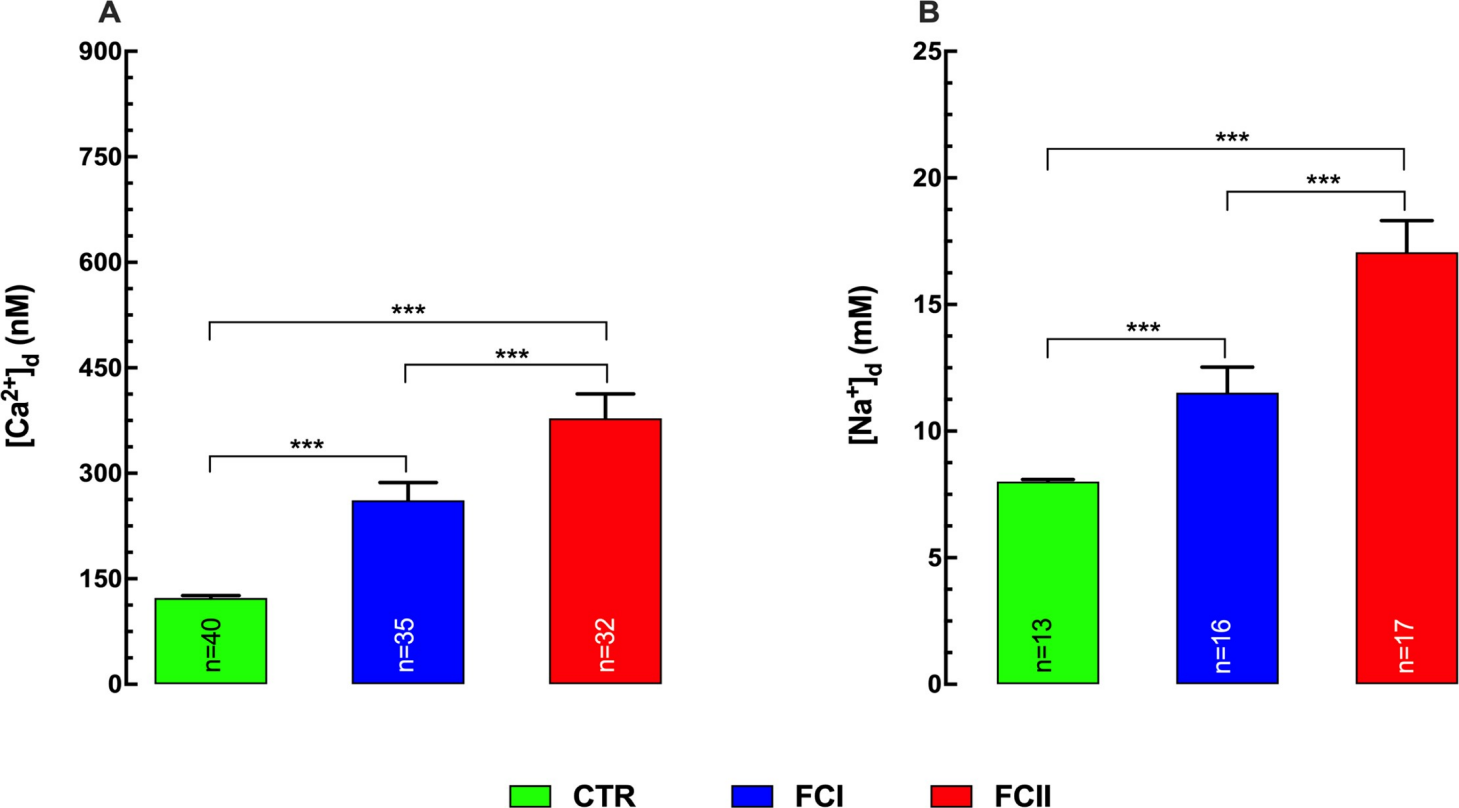
Diastolic [Ca^2+^] and [Na^+^] are increased in Chagas’ cardiomyocytes. Summary of the recording of [Ca^2+^]_d_ (2A) and [Na^+^]_d_ (2B) in cardiomyocytes isolated from control and FCI and FCII Chagas’ cardiomyocytes. Cardiomyocytes were obtained from 17 control individuals, 18 Chagas’ FCI, and 14 Chagas’ FCII patients. ***n*** represents the number of cardiomyocytes in which a successful measurement of [Ca^2+^]_d_ was carried out. Data are expressed as means ± S.D. Statistical analysis was performed using one-way ANOVA, followed by Tukey’s multiple comparison tests, *** p≤0.001.

We previously observed a significant increase in [Ca^2+^]_d_ in CC patients, which correlate directly with the extent of their cardiac dysfunction (NYHA class) regardless of gender [23]. Fig 1A, 1B and 1C are representative records showing simultaneous measuring of the resting membrane potential and [Ca^2+^]_d_ in single cardiomyocyte isolated from control (A), FCI (B), and FCII (C) Chagas’ cardiomyocytes. An elevation of [Ca^2+^]_d_ and a partial depolarization were observed in cardiomyocytes isolated from FCI and FCII Chagas patients. In control cardiomyocytes [Ca^2+^]_d_ was 123±3 nM (n = 40), while that in CC patients from FCI patients [Ca^2+^]_d_ was 262±25 nM (n = 35) (p≤0.001 compared to control), and in cardiomyocytes from FCII patients [Ca^2+^]_d_ was 378±34 nM (n = 32) (p≤0.001 compared to control and FCI) (Fig 2A). No gender difference in [Ca^2+^]_d_ was observed between FCI and FCII the Chagas’ patients_._ The partial depolarization observed in cardiomyocytes isolated from Chagas’ patients correlates with the level of cardiac dysfunction determined by the NYHA classification. We found a 6% reduction in average Vm values in cardiomyocytes from FCI patients, and 11% in cardiomyocytes from FCII patients compared to control. These results confirm and extend our previous report demonstrating a diastolic Ca^2+^ dysfunction in human cardiomyocytes from patients with Chagas’ disease [23].

A significant difference for [Na^+^]_d_ was observed in cardiomyocytes isolated from FCI and FCII Chagas patients compared to control. In control [Na^+^]_d_ was 8±0.1 mM (n = 13) compared to 12±1 mM (n = 16) and 17±1.2 mM (n = 17) in FCI and FCII cardiomyocytes respectively (p≤0.001 compared to control) (Fig 2B). These results demonstrate that there is a diastolic Ca^2+^ and Na^+^ overload in chagasic cardiomyocytes compared to control cells.

### IP_3_ effects on [Ca^2+^]_d_

**Fig 3:**
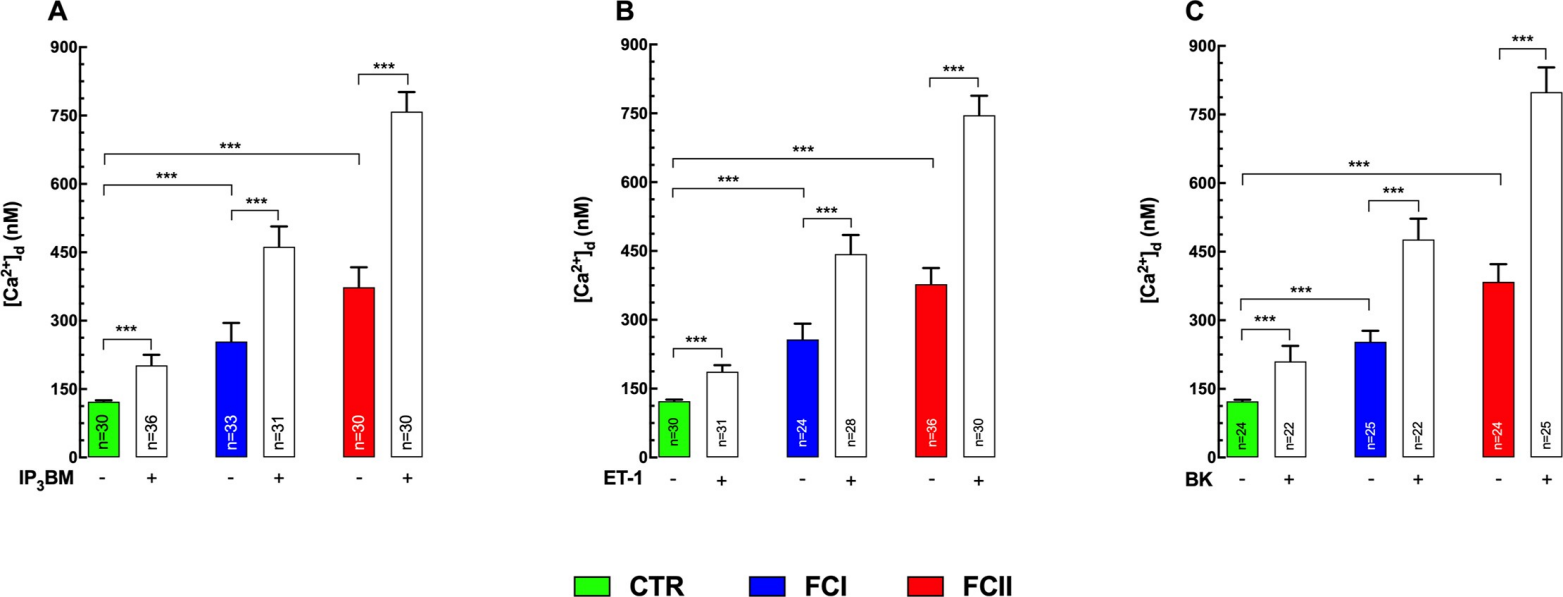
Effects of ET-1, BK and IP_3_BM on [Ca^2+^]_d_ in cardiomyocytes from control and Chagas’ patients. [Ca^2+^]_d_ was measured using Ca^2+^-selective microelectrodes before and after treatments with agents that enhance intracellular inositol 1,4,5 trisphosphate generation or concentration. **(A)** Effects of 10 μM membrane-permeant myoinositol 1,4,5-trisphosphate hexakis(butyryloxy-methyl) ester (IP_3_BM) on [Ca^2+^]_d_ in cardiomyocytes isolated from control (CTR), FCI, and FCII patients. **(B)** Effects of 100 nM endothelin (ET-1) on [Ca^2+^]_d_ in cardiomyocytes from control, FCI, and FCII patients. **(C)** Effects of 10 nM bradykinin (BK) on [Ca^2+^]_d_ in cardiomyocytes from control individuals, FCI, and FCII patients. Cardiomyocytes were obtained from 9–12 control individuals, 9–11 Chagas’ FCI, and 6–10 Chagas’ FCII patients respectively. ***n*** represents the number of cardiomyocytes in which a successful measurement of [Ca^2+^]_d_ was carried out. Data are expressed as means ± S.D. Statistical analysis was performed using one-way ANOVA, followed by Tukey’s multiple comparison tests, *** p≤0.001.

The role of IP_3_ in cardiomyocytes from Chagas’ patients was further studied using the membrane-permeant myoinositol 1,4,5-trisphosphate hexakis(butyryloxy-methyl) ester (IP_3_BM). IP_3_BM evokes the pharmacological effect of IP_3_ directly, avoiding the effects of phospholipase C activation [31]. 10 μM IP_3_BM elicited a robust increase in [Ca^2+^]_d_ in both control and Chagas’ cardiomyocytes, but the elevation was greater in the cardiomyocytes isolated from Chagas’ patients than control (FCII>FCI>control) (Fig 3A). IP_3_BM elevated [Ca^2+^]_d_ from 122±3 nM (n = 30) to 202±22 nM (n = 36) (p≤0.001), while in FCI-cardiomyocytes [Ca^2+^]_d_ rose from 255±40 nM (n = 33) to 462±44 nM (n = 31) (p≤0.001). In FCII-cardiomyocytes, [Ca^2+^]_d_ increased from 374±43 nM (n = 30) to 759±43 nM (n = 30) (p≤0.001) (Fig 3A). Incubation at higher [IP_3_BM] (up to 30 μM) still evoked a differential pharmacological effect on [Ca^2+^]_d_ between Chagas’ and control cardiomyocytes. The incubation in L-myoinositol 1,4,5-trisphosphate hexakis(propionyloxy-methyl) ester (L-IP_3_PM) did not induce changes in [Ca^2+^]_d_ either in control or CC indicating that the action of the ester was highly specific (S1 Fig). The Ca^2+^ elevation induced by IP_3_BM was not modified by removal of extracellular Ca^2+^ (see **Extracellular Ca**^**2+**^ **contribution**)

### Effect of Endothelin-1 on [Ca^2+^]_d_

Endothelin (ET-1) is a peptide that increases endogenous [IP_3_] and causes IP_3_-dependent Ca^2+^ release [39, 40] and has been implicated in the pathogenesis of CC [41, 42]. Thus, to investigate more fully the role of IP_3_ in the pathogenesis of Chagas’ heart disease cardiomyocytes from control and Chagas’ patients were exposed to ET-1 and [Ca^2+^]_d_ determined. Incubation in 100 nM ET-1 for 15 min induced an increase in [Ca^2+^]_d_ that was significantly higher in Chagas’ than in control cells (FCII>FCI>control) (Fig 3B). In control cardiomyocytes, incubation with ET-1 elicited an elevation of [Ca^2+^]_d_ from 123±3 nM (n = 30) to 187±14 nM (n = 31) (p≤0.001 compared to untreated control). In Chagas’ cardiomyocytes from FCI hearts [Ca^2+^]_d_ rose from 258±34 nM (n = 24) to 443±42 nM (n = 28) (p≤0.001 compared to untreated cardiomyocytes). In cardiomyocytes isolated from FCII patients, it increased from 378±35 nM (n = 36) to 746±42 nM (n = 30) (p≤0.001 compared to untreated cardiomyocytes) (Fig 3B). The Ca^2+^ elevation induced by ET-1 was not inhibited by the removal of extracellular Ca^2+^ in control or Chagas’ FCI and FCII cardiomyocytes (see **Extracellular Ca**^**2+**^ **contribution**).

### Bradykinin elevates [Ca^2+^]_d_

To further test the role of IP_3_, we investigated the effects of bradykinin (BK), a peptide that induces IP_3_ and diacylglycerol formation in cardiomyocytes through activation of the G-protein-coupled receptor and phospholipase C (PLC) [43] which has been implicated in the pathogenesis of CC [44]. Incubation of control and Chagas’ cardiomyocytes in 10 nM of BK for 10 min elevated [Ca^2+^]_d_ in all cells analyzed. However, the increase in [Ca^2+^]_d_ was greater in Chagas’ than in control cardiomyocytes (FCII>FCI>control) (Fig 3C). BK raised [Ca^2+^]_d_ from 123±4 nM (n = 24) to 210±33 nM (n = 22) in control cardiomyocytes (p≤0.001 compared to untreated cardiomyocytes). In FCI cardiomyocytes, [Ca^2+^]_d_ was increased from 253±24 nM (n = 25) to 477±45 nM (n = 22) (p≤0.001 compared to untreated cardiomyocytes). In FCII cardiomyocytes, [Ca^2+^]_d_ rose from 385±38 nM (n = 24) to 799±54 nM (n = 25) *(*p≤0.001 compared to untreated and FCI cardiomyocytes) (Fig 3C). The omission of extracellular Ca^2+^ did not modify the BK effect on [Ca^2+^]_d_ in control or Chagas’ cardiomyocytes (see **Extracellular Ca**^**2+**^ **contribution**)_._

### Xestospongin C partially restores [Ca^2+^]_d_

**Fig 4:**
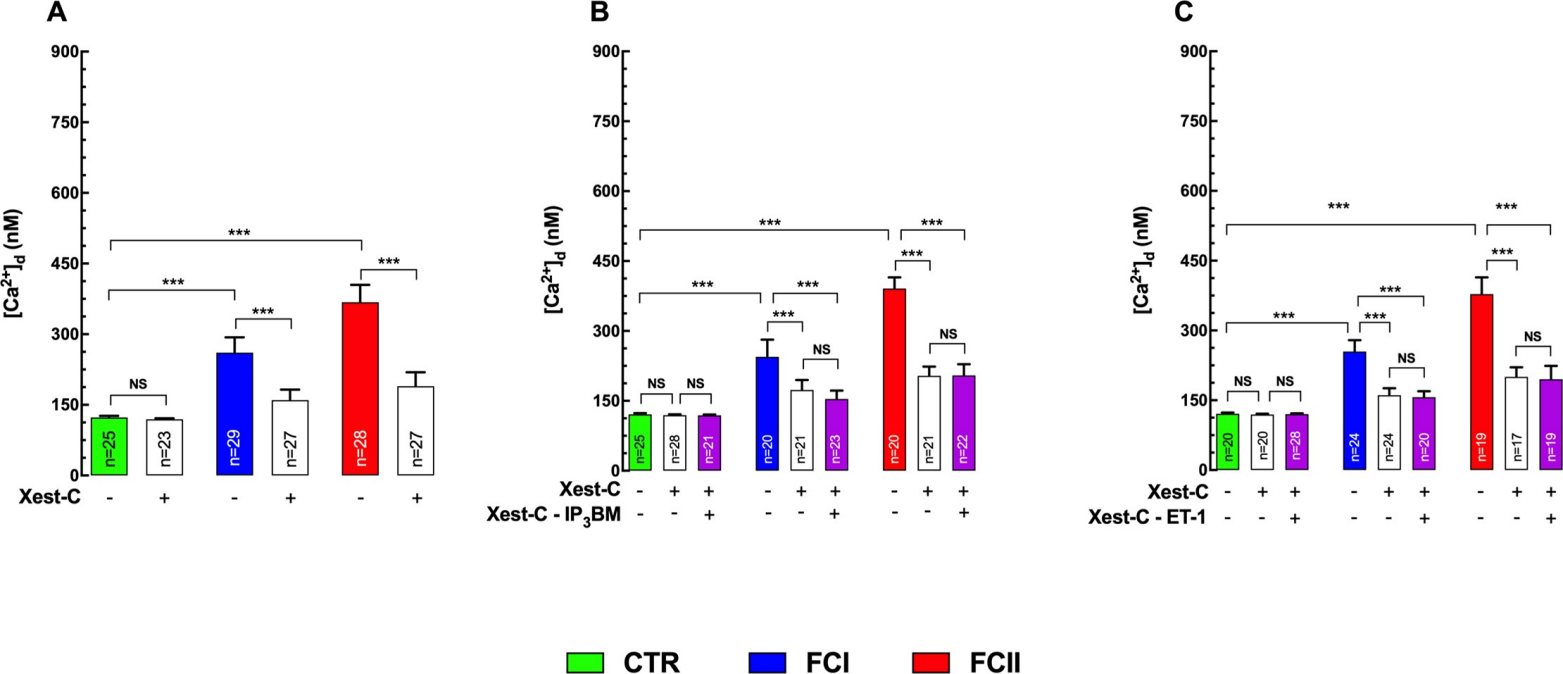
Xestospongin-C reduces [Ca^2+^]_d_ and prevents the effects of IP_3_BM, and ET-1 on diastolic Ca^2+^ concentration. [Ca^2+^]_d_ was measured in cardiomyocytes isolated from control (CTR) and Chagas patients (FCI and FCII). **(A)** Pretreatment with Xest-C reduced significantly [Ca^2+^]_d_ in FCI and FCII, but not in control cardiomyocytes_;_ **(B)** Incubation in Xest-C prevented the elevation of [Ca^2+^]_d_ induced by 10 μM myoinositol 1,4,5-trisphosphate hexakis(butyryloxy-methyl) ester (IP_3_BM) in all cells; **(C)** Xest-C inhibited the effect of 100 nM Endothelin (ET-1) on [Ca^2+^]_d_ in control and FCI and FCII cardiomyocytes. Cardiomyocytes were obtained from 9–10 control individuals, 10–12 Chagas’ FCI, and 8–10 Chagas’ FCII patients, respectively. Data are expressed as means ± S.D. Statistical analysis was performed using one-way ANOVA, followed by Tukey’s multiple comparison tests, *** p≤0.001.

The effects of xestospongin C (Xest-C), a membrane-permeable selective blocker of the IP_3_R [45], were investigated on the observed increase in diastolic Ca^2+^ in Chagas’ cardiomyocytes. [Ca^2+^]_d_ was measured before and after incubation for 15 minutes with 5 μM Xest-C. Treatment with Xest-C caused a significant reduction in [Ca^2+^]_d_ in Chagas’ cardiomyocytes but not in control cardiomyocytes (123±3 nM (n = 25) versus 120±2 nM (n = 23) (p>0.05 compared to untreated cells) (Fig 4A)_._ In cardiomyocytes isolated from FCI Chagas’ patients, [Ca^2+^]_d_ fell from 261±32 nM (n = 29) to 160±23 nM (n = 27) (p≤0.001 compared to untreated cardiomyocytes), and in FCII cardiomyocytes [Ca^2+^]_d_ decreased from 368±37 nM (n = 28) to 190±29 nM (n = 27) (p≤0.001 compared to untreated and FCI cardiomyocytes) (Fig 4A). The effect of Xest-C on [Ca^2+^]_d_ in Chagas’ cardiomyocytes was reversed by continuous washout from the bath (at least 15 minutes). Furthermore, Xest-C prevented the elevation of [Ca^2+^]_d_ in control and Chagas’ cardiomyocytes elicited by IP_3_BM (Fig 4B) and ET-1 (Fig 4C) (p>0.05 compared to untreated cells).

### Sarcoplasmic reticulum Ca^2+^ loading

**Fig 5:**
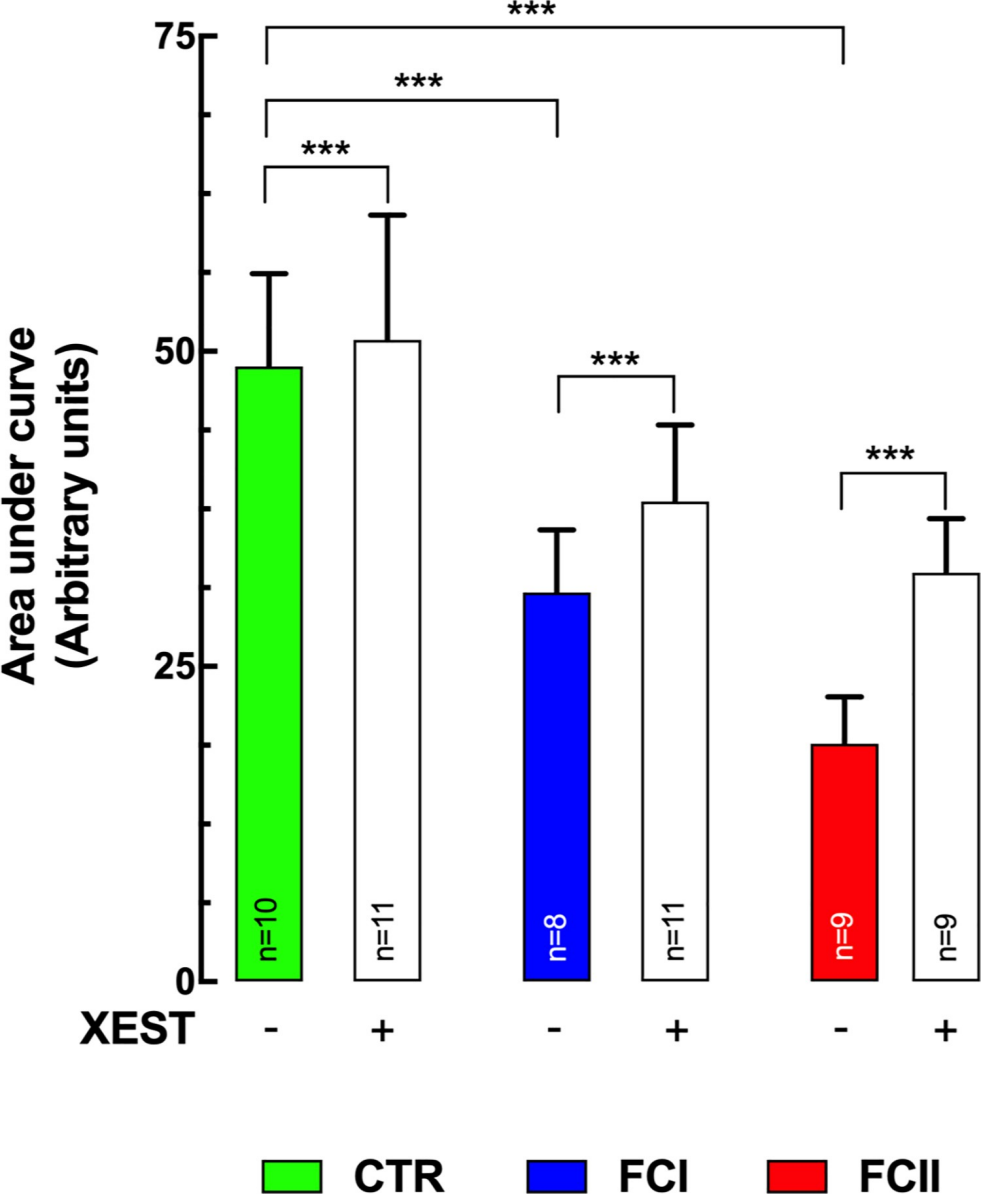
Decreases sarcoplasmic reticulum Ca^2+^ loading in chagasic cardiomyocytes. Control (CTR) and Chagas’ cardiomyocytes loaded with Fluo-4-AM were exposed to caffeine in Ca^2+^-free solution. Under these conditions, the total Ca^2+^ released was significantly smaller in *Chagas’* cardiomyocytes (FCI<FIC) compared with the control cardiomyocytes (area under the curve: 49±7 in control *versus* 31±5 (p≤0.001) in FCI and 19±4 (p≤0.001) in FCII). Cardiomyocytes were obtained from 6 control individuals, 7 Chagas’ FCI, and 8 Chagas’ FCII patients respectively. Data are expressed as means ± S.D. Statistical analysis was performed using one-way ANOVA, followed by Tukey’s multiple comparison tests, *** p≤0.001.

The level of the SR Ca^2+^ store was determined by exposing Fluo-4-AM loaded-control and Chagas’ cardiomyocytes to 10 mM caffeine [33]. Under these conditions, the total Ca^2+^ released was significantly smaller in Chagas’ cardiomyocytes compared with control cardiomyocytes. Quantitative analysis of the Ca^2+^ signal indicates that the Ca^2+^ SR loading was 37% lower in FCI (n = 8) than control cardiomyocytes (n = 10) (p≤0.001), and in the FCII was reduced by 61% (n = 9) (p≤0.001) (Fig 5). Moreover, treatment with 5 μM Xest-C for 15 min, partially restored the SR Ca^2+^ content in FCI and FCII Chagas’ cardiomyocytes (Fig 5). SR Ca^2+^ content was increased by 25% in FCI (n = 11) (p≤0.001 compared to untreated cells) and by 71% in FCII (n = 9) (p≤0.001 compared to untreated cells) in Chagas’ cardiomyocytes. No significant difference was observed in control cardiomyocytes after Xest-C treatment (n = 11) (p>0.05). These results suggest that the reduction in the SR Ca^2+^ levels appears to be mediated by an IP_3_Rs-Ca^2+^ leak from the SR.

### Intracellular [IP_3_]

**Fig 6:**
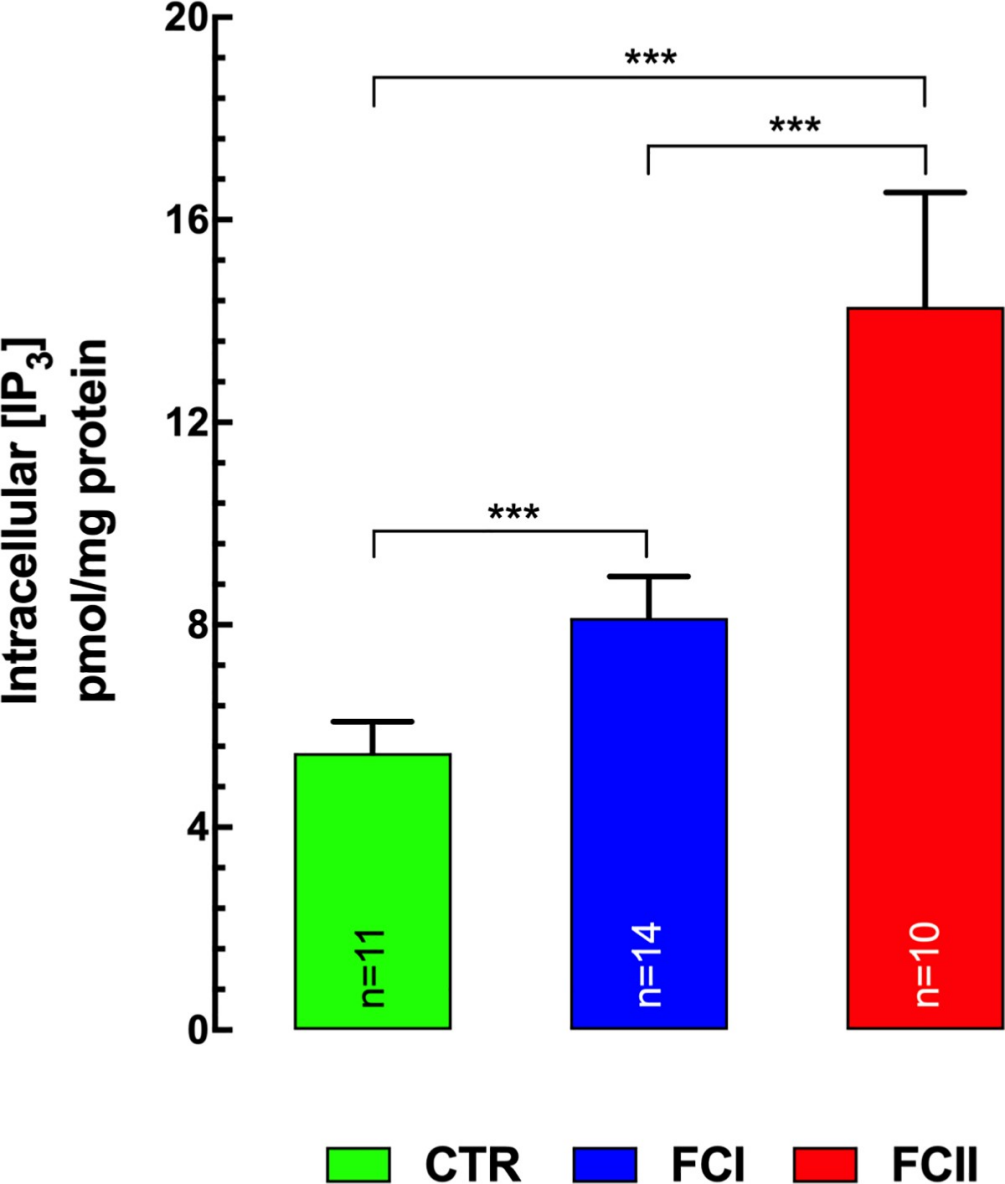
Intracellular [IP_3_] in control and Chagas’ cardiac tissue. Levels of [IP_3_]_i_ were determined by the competitive radioligand-binding assay. [IP_3_]_i_ was significantly higher in ventricular cells in patients with Chagas’ disease (FCI and FCII) than in control (CTR). [IP_3_]_i_ was elevated by 47% in FCI and 174% in FCII compared with control hearts. Heart samples were obtained from 10 control individuals, 11 FCI, and 10 FCII patients, respectively. ***n*** represents the number of determinations. Data are expressed as means ± S.D. Statistical analysis was performed using one-way ANOVA, followed by Tukey’s multiple comparison tests, *** p≤0.001.

Levels of intracellular IP_3_, as determined by the competitive radioligand-binding assay were significantly higher in ventricular cells in patients with Chagas’ disease than in control. The basal level of [IP_3_]_i_ was 5.4±0.6 pmol/mg protein (n = 11) in control cardiomyocytes (Fig 6), while in Chagas’ cardiomyocytes classified as FCI [IP_3_]_i_ was 8.1±0.8 pmol/mg protein (n = 14) (p≤0.001 compared to control and FCII values) and in those classified as FCII [IP_3_]_i_ was 14±2 pmol/mg protein (n = 10) (p≤0.001 compared to control and FCI values) (Fig 6).

### Extracellular Ca^2+^ contribution

**Fig 7:**
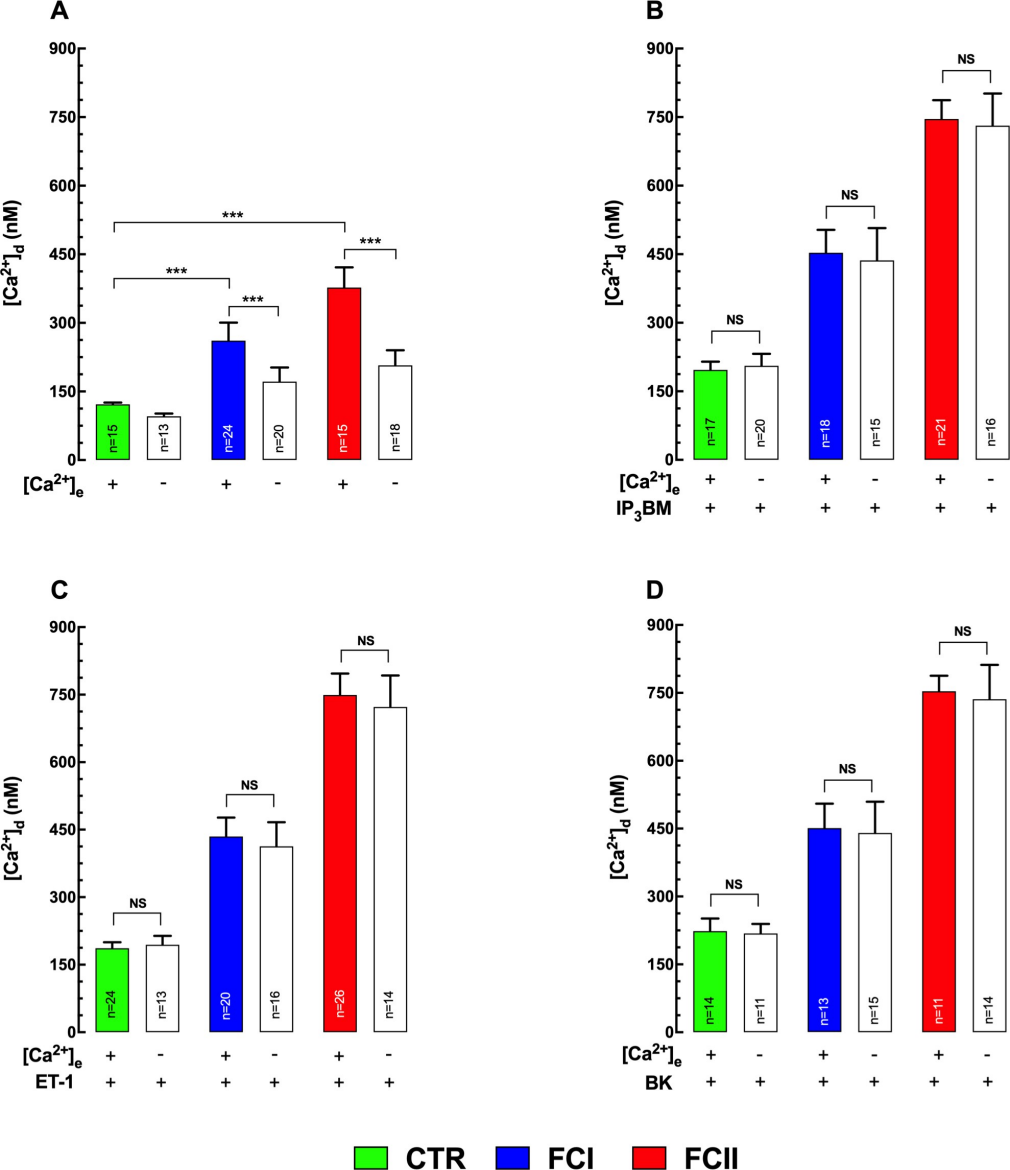
Effects of extracellular Ca^2+^ on [Ca^2+^]_i_ in control and Chagas’ cardiomyocytes. **(A)** Incubation of cardiomyocytes in Ca^2+^-free medium (see Materials and Methods) resulted in a significant reduction in [Ca^2+^]_d_ in both groups of cells. However, the magnitude of [Ca^2+^]_d_ decrease was more significant in Chagas (FCII>FCI) compared to the control (CTR) cardiomyocytes; **(B)** Removal of extracellular Ca^2+^ did not block the effect of IP_3_BM on [Ca^2+^]_d_ in control and Chagas’ cardiomyocytes_._ **(C)** Withdrawal of extracellular Ca^2+^ did not inhibit the effect of ET-1 on [Ca^2+^]_d_ in control and Chagas’ cardiomyocytes. **(D)** The effect of BK on [Ca^2+^]_d_ in control and Chagas’ cardiomyocytes was not modified by free Ca^2+^ solution. Cardiomyocytes were obtained from 6 control individuals, 8 Chagas’ FCI, and 6 Chagas’ FCII patients**. *n*** represents the number of cardiomyocytes in which a successful measurement was carried out; Data are expressed as means ± S.D. Statistical analysis was performed using one-way ANOVA, followed by Tukey’s multiple comparison tests, ** p≤0.01, *** p≤0.001.

To investigate the possible involvement of extracellular Ca^2+^ in the elevated [Ca^2+^]_d_ observed in Chagas’ cardiomyocytes, we conducted experiments in Ca^2+^-free medium (see Materials and Methods). Incubation of cardiomyocytes in a Ca^2+^-free medium for 5 minutes resulted in a significant reduction in [Ca^2+^]_d_ in all cardiomyocytes. The magnitude of [Ca^2+^]_d_ decrease was more significant in Chagas compared to control cardiomyocytes. In control cardiomyocytes [Ca^2+^]_d_ decreased from 122±4 nM (n = 15) to 96±6 nM (n = 13) (p≤0.001 compared to untreated cells), In FCI from 261±39 nM (n = 24) to 172±31 nM (n = 20) (p≤0.001 compared to untreated cells) and in FCII from 377±44 nM (n = 15) to 207±33 nM (n = 18) (p≤0.001 compared to untreated cells) (Fig 7A). Removal of extracellular [Ca^2+^] did not modify significantly the effect of IP_3_BM, ET-1, and BK on [Ca^2+^]_d_ in Chagas’ and control cardiomyocytes (Fig 7B, 7C and 7D) (p>0.05). These data indicate that the robust elevation of [Ca^2+^]_d_ elicited by IP_3_BM, ET-1, and BK in Chagas’ and control cardiomyocytes is coming from an intracellular store rather than an extracellular Ca^2+^ influx. Furthermore, that Ca^2+^ entry from extracellular space plays a role in the perturbed cytosolic Ca^2+^ regulation observed Chagas’ cardiomyocytes.

### Contractile functions of Chagas’ cardiomyocytes

**Fig 8:**
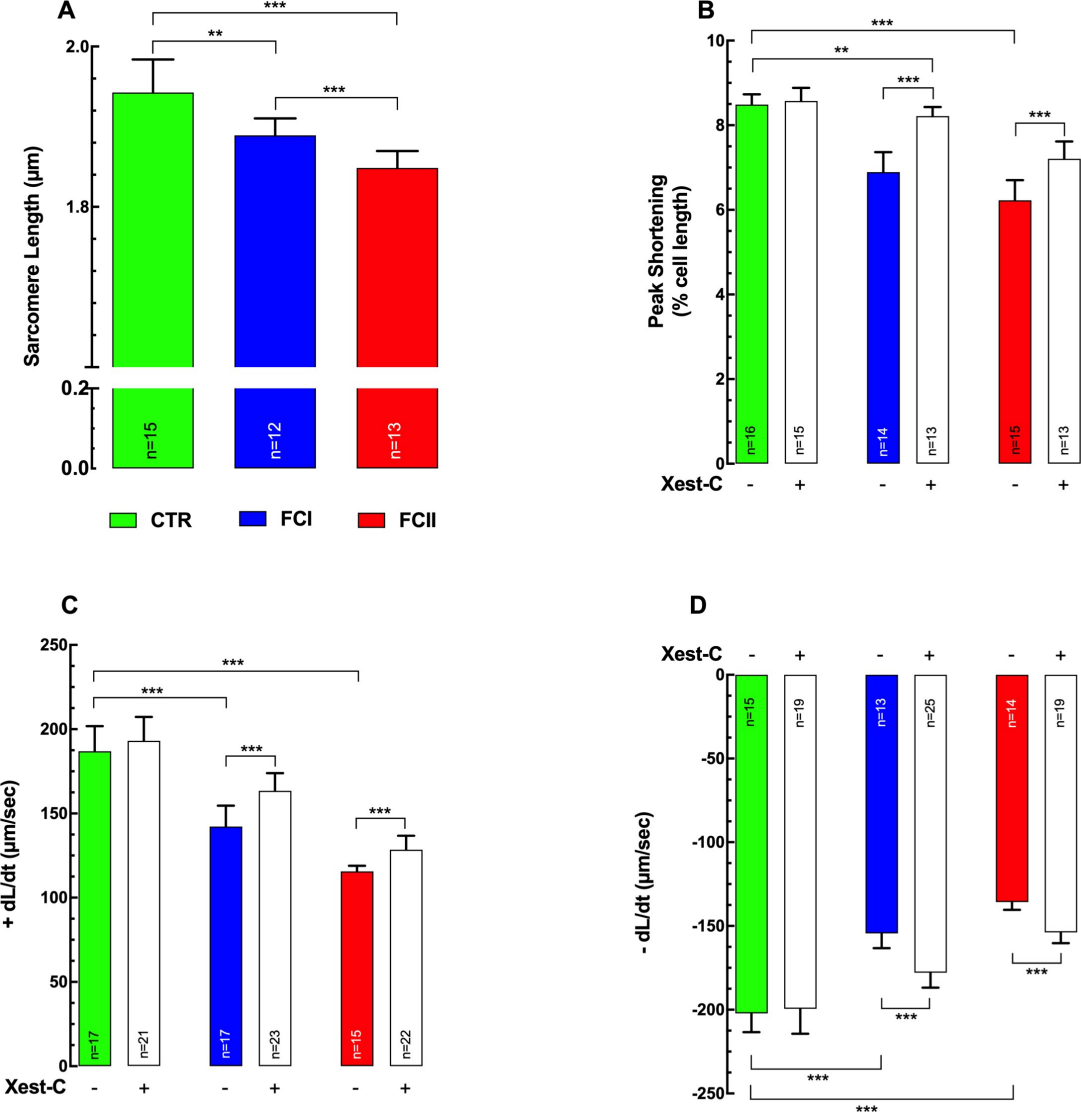
The depressed contractile function of Chagas’ cardiomyocytes is improved by Xestospongin-C treatment. Cardiomyocytes were isolated from control (CTR) and Chagas patients (FCI and FCII) and observed using a video-based edge-detection system. **(A)** Resting sarcomere length was determined following 30 s of field stimulation at a frequency of 1 Hz (2 ms pulse duration, ~1.5x threshold voltage) in quiescent cardiomyocytes; **(B)** Peak shortening (PS), **(C)** maximal velocity of shortening (+dL/dt), and (**D)** maximal velocity of relengthening (−dL/dt) were determined using steady-state twitches from 1 Hz electrical stimulation (2 ms pulse duration, ~1.5x threshold voltage). Cardiomyocytes were obtained from 7–9 control individuals, 9–11 Chagas’ FCI, and 7–9 Chagas’ FCII patients. ***n*** represents the number of cardiomyocytes in which a successful measurement was carried out. Data are expressed as means ± S.D. Statistical analysis was performed using one-way ANOVA, followed by Tukey’s multiple comparison tests. ** p≤0.01, *** p≤0.001.

Heart failure is the most significant and severe manifestation of human CC [46]. We found Chagas’ cardiomyocytes show depressed contractile properties versus control cardiomyocytes across all parameters studied. The average diastolic sarcomere length was significantly different between control and Chagas’ cardiomyocytes (1.94±0.04 μm, n = 15 for control *vs*. 1.89±0.02 μm, n = 12 and 1.85±0.02 μm, n = 13 for FCI and FCII respectively (p≤0.001 compared to control and p≤0.01 compared FCI *versus* FCII) (Fig 8A). The peak shortening (PS), the maximal velocity of shortening (+dL/dt), and maximal velocity of relengthening (-dL/dt) were decreased in FCI and in FCII cardiomyocytes compared to control (control>FCI>FCII) (Fig 8B, 8C and 8D). PS was decreased from 8.5±0.2% (n = 16) in control to 6.9±0.5% (n = 14) (p≤0.001) in FCI and to 6.2±0.4% (n = 15) (p≤0.001) in FCII cardiomyocytes (Fig 8B). +dL/dt was reduced from 187±15 μm/sec (n = 17) in control to 143±12 μm/sec (n = 17) (p≤0.001) in FCI and to 116±3 μm/sec (n = 15) (p≤0.001) in FCII (Fig 8C). Furthermore,–dL/dt also was decreased from 202±11 μm/sec (n = 15) in control to 154±8.7 μm/sec (n = 13) (p≤0.001) in FCI and to 136±5 μm/sec (n = 14) (p≤0.001) in FCII cardiomyocytes (Fig 6D). Xest-C does modify the contractile dysfunction in Chagas cardiomyocytes by significantly increasing: i) PS (23% in FCI and 16% in FCII cardiomyocytes), ii) +dL/dt (15% in FCI and 11% in FCII cardiomyocytes), and iii)–dL/dt (15% in FCI and 13% in FCII cardiomyocytes)(Fig 8B, 8C and 8D). It must be pointed out that Xest-C did not modify any of the parameters studied in control cardiomyocytes.

## Discussion

The current study reinforces our previous finding that a progressive deterioration of cardiac function in CC is associated at the cellular level with a defective intracellular Ca^2+^ regulation. CC is the most severe and life-threatening manifestation of human Chagas disease and is one of the most common causes of heart failure and sudden death in Latin America. This disease has become a public health concern that is not limited to populations in Latin America but also poses a global problem because of migration of infected individuals for economic and/or political reasons to developed countries, mainly Europe and the United States.

The present study confirms that human cardiomyocytes isolated from Chagas’ patients have an increase in [Ca^2+^]_d_ and a partial membrane potential depolarization, which corresponds with the degree of cardiac dysfunction determined by the NYHA classification [23]. In this report, we demonstrated, for the first time that IP_3_R activators, e.g., IP_3_BM, ET-1, and BK-induced a greater elevation of [Ca^2+^]_d_ in Chagas’ compared to non-Chagas’ human cardiomyocytes, which was not modified by the removal of [Ca^2+^]_e_. Furthermore, Chagas’ cardiomyocytes had a reduced SR Ca^2+^ loading and a higher level of intracellular IP_3_ with compromised contractile properties compared to control. Treatment with Xest-C, an IP_3_R blocker, improves [Ca^2+^]_d,_ increased SR-Ca^2+^ loading and ameliorates contractile dysfunction in Chagas’ cardiomyocytes.

Calcium is a central player in the regulation of cardiac contractility, and several cardiac pathologies have been associated directly or indirectly with changes in intracellular Ca^2+^ handling. Normal functioning of multiple mechanisms like plasma-membrane exchanger (Na^+^/Ca^2+^ exchanger) and pumps (PMCa^2+^ and SERCA-ATPase pumps) which control Ca^2+^ influx-efflux and reuptake allow for maintaining proper [Ca^2+^]_d_ during the rest period of the cardiac cycle (diastole) within a physiological range (~100 nM) [23, 34]. The [Ca^2+^]_d_ values obtained from the control cardiomyocytes concur with previous estimations of the diastolic Ca^2+^ level in human ventricular myocytes using Ca^2+^-selective microelectrodes [23] and fluorescent Ca^2+^ indicator fluo-3 [47–49]. The magnitude of diastolic Ca^2+^ elevation observed in Chagas cardiomyocytes correspond with the patients’ functional class (NYHA). Perturbed intracellular Ca^2+^ regulation in Chagas cardiomyocytes favors an intracellular Ca^2+^ overload with direct consequences to systolic and diastolic function and also promotes arrhythmias, which have observed in patients suffering from CC [23, 50, 51]. Furthermore, chronic elevations in [Ca^2+^]_d_ as observed in Chagas’ cardiomyocytes is deleterious to muscle cell function because increase calpain activation and impairment of autophagy and mitochondrial function [52, 53].

The changes in the [Ca^2+^]_d_ found in Chagas’ cells are qualitatively similar to those reported in human epithelial cells infected with *T*. *cruzi* [54]. We consider that the elevation of [Ca^2+^]_d_ observed in Chagas’ patients is related to the CC and not a resultant side effect from the patient’s pharmacological treatment because all medications were suspended 48 h before the endomyocardial biopsy_._ The observed partial depolarization in Chagas’ cardiomyocytes from FCI and FCII patients may relate to a diastolic Na^+^ overload found in human Chagas’ cardiomyocytes (1.4-fold in FCI and 2.1-fold in FCII compared to control). A membrane depolarization associated with intracellular Na^+^ overload has been described in skeletal muscle cells [55]. Besides, an elevated [Na^+^]_d_ can contribute to a further intracellular Ca^2+^ overload through the reverse mode of sarcolemmal Na^+^/Ca^2+^ exchanger [56].

We previously presented evidence of a possible link between Chagas’ infections and altered cellular Ca^2+^ homeostasis and the intracellular messenger IP_3_ [23]. Treatment with U-73122, a ß-phospholipase C inhibitor, and 2-APB partially reduced the elevated [Ca^2+^]_d_ in the Chagas’ cardiomyocytes [23]. IP_3_-dependent Ca^2+^ release represents the major pathway of intracellular Ca^2+^ release in electrically non-excitable cells [24]. Although type 1 and 2 IP_3_ receptors have been identified in several areas of cardiac cells and an IP_3_-Ca^2+^ release has been well documented [57], the role of IP_3_ in excitation-contraction coupling and cardiac function in the mammalian heart has remained controversial [58]. Several studies suggest that IP_3_ may be involved in the regulation of the gene transcription [59], the amplification of ryanodine receptor signals [60], and the regulation of Ca^2+^ influx through the modulation of transient receptor potential channel (TRPC) [34]. In contrast to the physiological condition, a more pronounced role of IP_3_ has been suggested under various cardiac pathologies (e.g., cardiac hypertrophy, ischemic dilated cardiomyopathy, atrial fibrillation, failing myocardium and hypertension) [26, 61, 62]. Thus, increased expression of IP_3_Rs in the perinuclear compartment has been observed hypertrophied and failing hearts, which have associated with altered nucleoplasmic Ca^2+^ regulation and an increase in diastolic [Ca^2+^]_d_ [63]. In this context, Harzheim et al. [25] have suggested that an increase in IP_3_Rs expression is a general mechanism that underlies remodeling of Ca^2+^ signaling during heart disease, and in particular, in triggering arrhythmia during hypertrophy. Moreover, IP_3_-induced Ca^2+^ release is increased in SR microsomes prepared from hypertrophic myocytes [64]. Additionally, elevated IP_3_R levels and increased InsP_3_ binding has been reported in the left ventricle during human heart failure [29].

Further support for the IP_3_ involvement in CC was obtained by showing that exposure of cardiomyocytes to agents that enhance endogenous generation or concentration of IP_3_ like IP_3_BM, ET-1 or BK [39, 40] caused an elevation in [Ca^2+^]_d_ which was always greater in cardiomyocytes from Chagas’ patients than non-Chagas’ subjects and related to the degree of cardiac dysfunction (FCII>FCI). The differential pharmacological effect of IP_3_BM on [Ca^2+^]_d_ in Chagas’ cardiomyocytes persists up to a concentration of 30 μM, where the [IP_3_]_i_ levels would be equivalent between control and Chagas’ cardiomyocytes, suggesting a greater IP_3_Rs expression in Chagas cardiomyocytes compared to control. The IP_3_BM, ET-1, or BK effects on [Ca^2+^]_d_ were not modified by the removal of extracellular Ca^2+^, but it was inhibited by Xest-C, suggesting that their pharmacological action is mediated through IP_3_-dependent Ca^2+^ release. These results reinforce the notion that increased [Ca^2+^]_d_ observed in Chagas’ cardiomyocytes is mediated in part by activation of IP_3_Rs.

The fact that incubation in L-IP_3_PM did not induce any change in [Ca^2+^]_d_ either in control or Chagas’ cardiomyocytes indicates that the action of IP_3_BM was highly specific. Individuals with CC had increased levels of ET-1 in plasma [42], plasma ET-1 levels are elevated in mice infected with *T*. *cruzi*, and there is an increased expression of myocardial mRNA for ET-1 [65]. These findings represent the first report of an IP_3_-enhanced release of intracellular Ca^2+^ induced by IP_3_BM-, ET-1-, or BK in human Chagas’ cardiomyocytes.

In Chagas’ cardiomyocytes, chronic elevated [Ca^2+^]_d_ may enhance the IP_3_ sensitivity of IP_3_Rs [66] and could well synergize with the other factors that further elevate [Ca^2+^]_d_. An increase in IP_3_Rs expression has been reported in atrial myocytes of humans and dogs during atrial fibrillation and in human heart failure [67, 68]. The IP_3_R expression is significantly elevated in rat cardiac tissue from aorta-banded hypertrophic mice and human ischemic heart with dilated cardiomyopathy [25, 26, 29]. An elevated IP_3_R expression may represent a plausible explanation for the increased [Ca^2+^]_d_ observed in Chagas’ cardiomyocytes.

In cardiomyocytes isolated from Chagas’ patients [IP_3_]_i_ was higher compared to those from control subjects. It has been previously shown in various types of cells that elevation of IP_3_ production, which release Ca^2+^ from intracellular stores [24, 69] may lead to an increase of [Ca^2+^]_d_ [69] and a robust Ca^2+^ release upon exposure to IP_3_BM-, ET-1-, or BK [69]. The elevated intracellular [IP_3_] can have two possible sources i) the plasma membrane of parasites in intracellular forms, such as amastigotes [70] and ii) IP_3_ derived from the plasma membrane of the host changes due to changes in IP_3_ synthesis and/or degradation [71, 72]. Furthermore, an elevated [IP_3_]_i_ may provoke an increase in Ca efflux from the SR, which could end in a depletion of intraluminal sarcoplasmic reticulum Ca^2+^ content [24, 73]. We have found in Chagas’ cardiomyocytes a decrease in SR Ca^2+^content compared to control (Control>FCI>FCII), and blocking the IP_3_Rs with Xest-C results in a significant increase in SR-Ca^2+^ content in Chagas’ cardiomyocytes which indicates that IP_3_Rs may play an intrinsic role in the intracellular Ca^2+^ dysregulation in CC.

Chagas’ cardiomyocytes exhibit markedly depressed contractile properties versus control across all parameters studied, such as peak shortening, maximal velocity of shortening (Control>FCI>FCII), which may be related to a reduced SR Ca^2+^ loading and subsequent intracellular Ca^2+^ release. It is well established that Ca^2+^ release directly regulates contractility of cardiomyocytes, and that a reduced release from intracellular stores decreases force development under heart failure [74, 75]. Chagas’ cardiomyocytes also showed an altered velocity of re-lengthening, which may be due to a defect of relaxation controlled by the SR-ATPase pump (SERCA), the NCX and/or the plasma membrane Ca^2+^ pump (PMCA). The chronic elevation of the intracellular IP_3_ levels in addition to the induced sustained increase in [Ca^2+^]_d_, also elicits a Ca^2+^ depletion of the SR, depressing the amount of Ca^2+^ for release upon electrical stimulation [24, 73]. Furthermore, a shorter resting sarcomere length was observed in Chagas’ cardiomyocytes, which corresponds with chronic elevated [Ca^2+^]_d_. Pretreatment with Xest-C partially reverse the contractile dysfunction in CC by significantly increasing PS, +dL/dt, and -dL/dt. The enhancement of contractile function induced by Xest-C may be related to the inhibition of IP_3_Rs and the prevention of SR Ca^2+^ depletion.

An interesting observation was that depletion of extracellular Ca^2+^ provoked a more significant reduction of [Ca^2+^]_d_ in Chagas than control cardiomyocytes. Several mechanisms of Ca^2+^ entry non-voltage dependent have been described in cardiac cells; among them, the TRPC, a diversely regulated family of plasma membrane permeable cation channels, which are activated by diacylglycerol, by depletion of intracellular Ca^2+^ stores or by stretch [76]. Biochemical and functional studies suggest a close coupling of some TRPC channels and InsP_3_R [77]. Further studies are necessary to establish the role of the TRPC channels in the CC.

In conclusion, patients suffering from CC have a chronic elevation of [Ca^2+^]_d_ that appears to be mediated by IP_3_Rs and is associated with the deterioration of cardiac function (FCII>FCI). Consistent with these results, agents that enhance intracellular IP_3_ generation like ET-1, BK, or membrane-permeant IP_3_ esters caused a further elevation in [Ca^2+^]_d_ more significant in cardiomyocytes from Chagas’ than non-Chagas’ subjects- and Xest-C an IP_3_Rs blocker decreased [Ca^2+^]_d_, and improved cardiomyocytes contractile response from Chagas’ patients. Furthermore, Chagas’ cardiomyocytes had a higher level of intracellular [IP_3_] with compromised SR-Ca^2+^ loading compared to control.

These novel findings reveal an unmask mechanism by which IP_3_ may play an essential role in the pathophysiology of CC and open the door for new therapeutic targets oriented at improving cardiac function and therefore, the quality of life of individuals suffering from CC. These discoveries are of paramount importance because there is still no highly effective cure available for those currently infected with *T*. *cruzi*, a third of which will develop potentially fatal cardiomyopathy.

### Limitations of the study

The major limitation of this study is that downstream IP_3_ cell-signaling and IP_3_Rs expressions in Chagas’ cardiomyocytes were not studied. Scarcity and accessibility to human endomyocardial tissue were restrictions to carry out those experiments. Endomyocardial biopsies are conducted in patients under sedation via fluoroscopic guidance, and the tissue samples from each patient studied are limited in size (2 to 3 mm^3^) and number (2 to 3 biopsies per patient). Furthermore, enzymatic isolation of intact ventricular cardiomyocytes from human heart biopsies is less successful than the retrograde perfusion of the whole heart used in experimental models. Determination of IP_3_ cell-signaling and the expression of IP_3_Rs in the human cardiac cells have been conducted in explanted hearts from patients who underwent cardiac transplantation [25, 29] or during coronary artery bypass surgery [68], where muscle size and tissue quantity are not limited. The observed changes in diastolic [Ca^2+^] and intracellular [IP_3_] in cardiomyocytes isolated from chagasic patients should be interpreted with caution. Both changes may occur as an epiphenomenon in a heart as a consequence of multiple pathological alterations observed in CC. However, despite the above limitations, we have confirmed the involvement of intracellular Ca^2+^ dysregulation, and we unmask a thus far unrecognized involvement of IP_3_ in the pathophysiology of CC.

## Supporting information

S1 Fig No effects of L-IP3PM on [Ca2+]d in cardiomyocytes from control and Chagas’ patients. [Ca^2+^]_d_ was measured using Ca^2+^-selective microelectrodes before and after treatments with L-myoinositol 1,4,5-trisphosphate hexakis(propionyloxy-methyl) ester (L-IP_3_PM). The incubation in L-IP_3_PM did not induce significant changes in [Ca^2+^]_d_ either in control (CTR) or Chagas’ cardiomyocytes. Cardiomyocytes were obtained from 8–10 control individuals, 7–9 Chagas’ FCI, and 6–8 Chagas’ FCII patients, respectively; ***n*** represents the number of cardiomyocytes in which a successful measurement of [Ca^2+^]_d_ was carried out. Data are expressed as means ± S.D. Statistical analysis was performed using one-way ANOVA, followed by Tukey’s multiple comparison tests, *** p≤0.001.
(TIF)
