# Supplementary figures and images for: Increases in [IP3]i aggravates diastolic [Ca2+] and contractile dysfunction in Chagas’ human cardiomyocytes
Source: PLoS Negl Trop Dis. 2020 Apr 10;14(4):e0008162. doi: 10.1371/journal.pntd.0008162 (PMC7176279; doi:10.1371/journal.pntd.0008162)

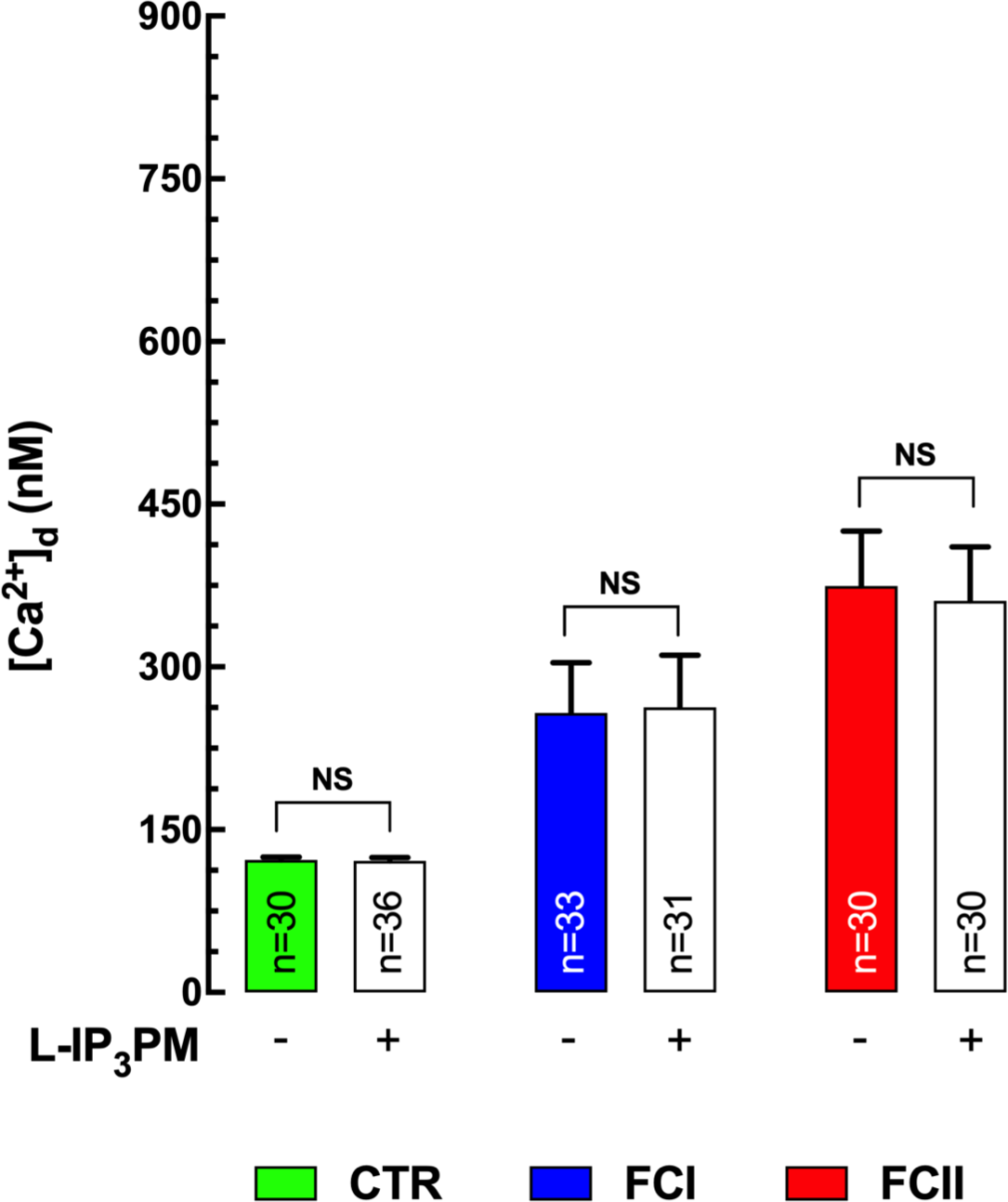

Supplement: S1 Fig — [Ca2+]d was measured using Ca2+-selective microelectrodes before and after treatments with L-myoinositol 1,4,5-trisphosphate hexakis(propionyloxy-methyl) ester (L-IP3PM). The incubation in L-IP3PM did not induce significant changes in [Ca2+]d either in control (CTR) or Chagas’ cardiomyocytes. Cardiomyocytes were obtained from 8–10 control individuals, 7–9 Chagas’ FCI, and 6–8 Chagas’ FCII patients, respectively; n represents the number of cardiomyocytes in which a successful measurement of [Ca2+]d was carried out. Data are expressed as means ± S.D. Statistical analysis was performed using one-way ANOVA, followed by Tukey’s multiple comparison tests, *** p≤0.001. (TIF) [file pntd.0008162.s001.tif]
